# Vagus Nerve Stimulation Decreases Pancreatitis Severity in Mice

**DOI:** 10.3389/fimmu.2020.595957

**Published:** 2021-01-14

**Authors:** Luyao Zhang, Zhiyang Wu, Zhihui Tong, Qi Yao, Ziyu Wang, Weiqin Li

**Affiliations:** ^1^ Department of Pathology, School of Medicine & Holistic Integrative Medicine, Nanjing University of Chinese Medicine, Nanjing, China; ^2^ Department of Critical Care Medicine, Qilu Hospital (Qingdao), Cheeloo College of Medicine, Shandong University, Qingdao, China; ^3^ Department of Critical Care Medicine, Research Institute of General Surgery, Jinling Hospital, Medical School of Nanjing University, Nanjing, China

**Keywords:** acute pancreatitis, vagus nerve stimulation, macrophage, α7nAChR, spleen

## Abstract

**Background:**

Vagus nerve stimulation (VNS) is effective in reducing inflammation in various diseases, such as rheumatoid arthritis, colitis and acute kidney injury. The anti-inflammatory effect of vagus nerve in these diseases necessitates the interactions of neural activation and α7 nicotinic acetylcholine receptors (α7nAChRs) on splenic macrophages. In this study, we aimed to investigate the effect of VNS on severity in experimental acute pancreatitis (AP).

**Methods:**

Two independent AP models were used, which induced in ICR mice with caerulein or pancreatic duct ligation (PDL). Thirty minutes after modeling, the left cervical carotid sheath containing the vagus nerve was electrically stimulated for 2 min. Plasma lipase and amylase activities, TNF-α levels and pancreas histologic damage were evaluated. In caerulein mice, the percentages of α7nAChR^+^ macrophage in pancreas and spleen were assessed by flow cytometry. Furthermore, splenectomy and adoptive transfer of VNS-conditioned α7nAChR splenocytes were performed in caerulein mice to evaluate the role of spleen in the protective effect of VNS.

**Results:**

VNS reduced plasma lipase and amylase activities, blunted the concentrations of TNF-α and protected against pancreas histologic damage in two AP models. Survival rates were improved in the PDL model after VNS. In caerulein AP mice, VNS increased the percentages of α7nAChR^+^ macrophages in pancreas and spleen. Adoptive transfer of VNS-treated α7nAChR splenocytes provided protection against pancreatitis in recipient mice. However, splenectomy did not abolish the protective effect of VNS.

**Conclusions:**

VNS reduces disease severity and attenuates inflammation in AP mice. This effect is independent of spleen and is probably related to α7nAChR on macrophage.

## Introduction

Acute pancreatitis (AP) is an inflammatory disease with different clinical courses, varying from self-limiting mild forms to life threatening severe AP (1). Almost half of AP deaths occur during the first two weeks and the majority of which is due to systemic organ failure (2). In the initial phase of AP, activated digestive enzymes cause pancreatic acinar cell injury. Acinar cells produce cytokines and chemokines, leading to neutrophils and macrophages recruitment. This results in further pancreas damage and the production of inflammatory cytokines such as IL-1, IL-6, and TNF-α (3). These mediators could cause systemic inflammatory response syndrome and ultimately remote organ injury. The events occurring after acinar cell injury are believed to determine the disease severity. Accumulating evidence suggests controlling inflammatory response is important in the early treatment of AP, which might affect the mortality.

Vagus nerve stimulation (VNS) was approved for clinical treatment of several conditions, including drug refractory epilepsy and depression (4, 5). Besides, VNS was suggested useful in controlling inflammation in rheumatoid arthritis and Crohn’s disease patients (6, 7). In experimental animals, VNS was found effective in sepsis, hemorrhagic shock and ischemia-reperfusion injury in brain, heart and kidney (8–10). It is commonly believed the immune modulation effect of VNS is through the cholinergic anti-inflammatory pathway (CAP) (11). When the vagus nerve is stimulated with electricity, the activated nerve in the celiac mesenteric ganglia releases acetylcholine (Ach), leading to the activation of adrenergic neurons of the splenic nerve. The splenic nerve releases noradrenaline, which communicates directly with T cells in the spleen. The activated T cells release another neurotransmitter, Ach. Ach binds to α7 nicotinic acetylcholine receptors (α7nAChRs) on macrophages, resulting in suppression of pro-inflammatory cytokines (12). However, while VNS has been efficacious in other models of human disease, it has not yet been demonstrated in pancreatitis.

The aim of our study was to investigate the effects of VNS on the pancreatitis severity and systemic inflammation in two mouse AP models and the specific roles of α7nAChR^+^ macrophage and spleen.

## Materials and Methods

### Mice

Wild-type mice (ICR; male; 25–30 g) were purchased from the Qing Longshan Animal Breeding Facility (Jiangning, Nanjing, China). All animal protocols were approved by the Institutional Animal Care and Use Committee of Nanjing University of Chinese Medicine and were performed in accordance with the guidelines for animal research.

### Caerulein-Induced AP Model and Pancreatic Duct Ligation (PDL) AP Model

For VNS optimization studies, a mild edematous pancreatitis was induced in mice by 10 hourly intraperitoneal injections (i.p.) of 100 μg/kg of caerulein (NJPeptide, Inc, Nanjing, China). For the remaining studies using the caerulein-induced AP model, same dose of caerulein was given hourly for 8 h. Normal control (NC) group received hourly i.p. injections of 0.3ml PBS. Two hours following the last injection, mice were sacrificed under general anesthesia and their blood was collected from the inferior vena cava inferior into sodium heparin-coated vacutainers. The pancreas and/or spleen were removed for flow cytometry and histological analysis.

PDL model, which mimic severe gallstone AP was performed (13). Briefly, mice were anesthetized with a mixture of ketamine (100 mg/kg) and xylazine (10 mg/kg) i.p. The duodenum was exposed and the distal common bile-pancreatic duct was ligated near its junction with the duodenum. Then the abdominal cavity was closed and mice were heated with a constant temperature of 37°C during recovery. Mice were sacrificed and samples were collected 12 h after PDL modeling.

### Vagus Nerve Stimulation and Survival Studies

Under the same anesthetize condition as PDL modeling, a ventral middle cervical incision was made and the salivary glands were separated and retracted laterally. The left carotid sheath was exposed and then isolated. The sheath containing the vagus nerve was placed on a bipolar hook electrode connected to an electrical stimulator (HANS, Nanjing, China). In this way, the left carotid sheath with the vagus nerve buried within would be stimulated as a whole part, which protects the vagus nerve from operation injuring. Half an hour after the first injection of caerulein or the PDL operation, electrical stimulation (a biphasic signal square waveform: cathodic first, 500 μs for a whole biphasic pulse, 225 μs for both phases and 50 μs duration for interphase delay) at 10 Hz was applied for 2 min long. In sham animals, the left carotid sheath was exposed and not stimulated.

For survival studies, surgical preparation was performed after PDL induction. With the left carotid sheath found, a bipolar cuff electrode (Kedoubc, Suzhou, China) with an internal diameter of 0.5 mm was implanted around the sheath as described by Crystal M. Noller et al. (14). The distance between contacts was 1 mm. The connecting wire was tunneled under the skin and the connector was externalized at the base of the neck. Electrical stimulation (a biphasic signal square waveform as described previously, 10 Hz, 0.3 mA, 2 min) was conducted at 30 min, 1 day and 3 days after PDL. Mice in the control group were implanted the same cuff electrode with no electrical stimulation. Mice that survived were observed for 8 days.

### Measurement of Evoked Potentials

Mice were anesthetized as previously described. A bipolar hook electrode was placed on the left carotid sheath. A midline abdominal incision was made and a bipolar hook electrode was placed across the ventral trunk of the subdiaphragmatic vagus nerve. The neural compound action potentials evoked by cervical VNS were measured with a filter of 160Hz-1KHz, 50Hz Notch and 10KHz sample rate (BL-420F, Taimeng, Chengdu, China). Waveform was rectified at stimulus rising edge and averaged by 30 times to reduce baseline noise.

### Adoptive Transfer Studies

Mice were anesthetized and submitted to VNS or sham treatment as previously described. Twenty-four h later the whole spleens were removed and passed through 40-μm filters into PBS to prepare single-cell suspensions. The cell pellet was collected by centrifugation (500 g, 5 min) and then erythrocytes were removed by Red Blood Cell lysis buffer (Biosharp, Hefei, China). After that, the samples were centrifuged and the cell pellet was diluted in PBS. And 1 × 10^6^ cells were injected *via* angular vein 1 day prior to AP.

### Splenectomy

Mice were anesthetized as previously described. The splenic vasculature was ligated and the spleen was removed. Mice in the sham splenectomy group underwent an abdominal small flank incision. Mice were allowed to recover for 5 days before AP model induction.

### Plasma Amylase, Lipase, and TNF-α Detections and Histological Evaluations of Pancreas and Lung

Levels of plasma amylase and lipase were measured with commercially available kits (BioSino Bio-Technology and Science Inc., Beijing, China; Jiancheng Corp., Nanjing, China). TNF-α levels in plasma and pancreas were analyzed by ELISA kits (MultiSciences, Hangzhou, China).

Pancreas and lung were obtained, fixed in neutral-buffered formaldehyde, embedded in paraffin, sectioned and stained with hematoxylin and eosin. Sections were analyzed blindly by a pathologist unaware of the groups. Pancreas damage was scored based on necrosis, inflammation, hemorrhage and edema (0–4 scale each) as previously described (15). A total severity score was calculated (maximum =16 points). Lung damage was evaluated for structure damage and inflammation (16).

### Isolation of Pancreatic Leukocytes and Splenocytes

Pancreatic leukocytes were obtained using a collagenase digestion method described previously for flow cytometry analysis (17). The pancreas was removed, minced in small fragments and digested in DMEM medium supplemented with 2% fetal bovine serum and collagenase IV (BioFroxx, Germany. at a concentration of 2mg/ml). Samples were incubated under agitation for 15 min at 37°C and vortexed at low speed for 20 s before passing through a 70-μm filter. Pancreas homogenates were submitted to centrifugation to obtain pancreas leukocytes.

The splenic tissue was teased apart and passed through a 70-μm filter. Erythrocytes were lysed and splenocytes were obtained.

### Flow Cytometry

Pancreatic leukocytes and splenocytes were stained with the following Abs: anti-CD45 APC (clone 30-F11, Biolegend, San Diego, CA, USA), anti-CD11b APC/Cy7 (clone M1/70, Biolegend, San Diego, CA, USA), anti-Ly6G FITC (clone RB6-8C5, eBioscience, CA, USA), anti-F4/80 PE/Cy7 (clone BM8, Biolegend, San Diego, CA, USA) and anti-α7nAChR PE (clone 319, Santa Cruz). Cells were suspended in Pharmingen Stain Buffer and analyzed using a ACEA NovoCyte flow cytometer with NoVo Express software (ACEA Biosciences, San Diego, CA, USA).

### Statistical Analysis

All data are expressed as mean± SE. Comparisons between groups were made using One-way ANOVA followed by Tukey test. Survival curves were derived by the Kaplan -Meier method and compared by the log-rank test. A p value < 0.05 was considered significant. All the analyses were performed with GraphPad Prism version 7 (GraphPad Software).

## Results

### The Threshold of Electrical Cervical Vagus Nerve Stimulation for Depolarizing the Vagus Nerve

**Figure 1:**
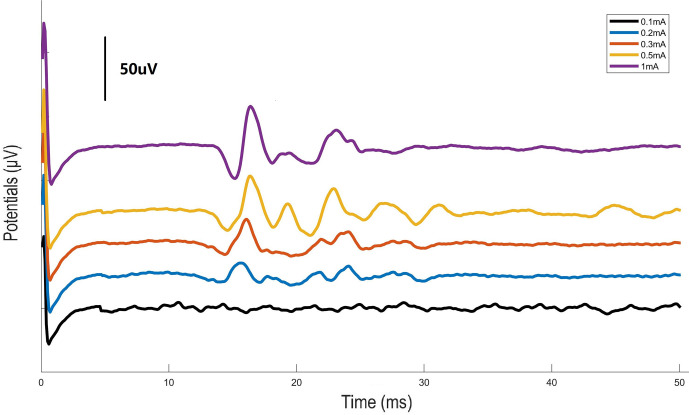
Graph illustrating the action potentials from the subdiaphragmatic vagus nerve. Electrical stimulation of the left carotid sheath with different intensities was performed and evoked potentials were measured at the subdiaphagmatic vagus nerve in mice.

Stimulation with the left carotid sheath activated compound action potentials from the subdiaphragmatic vagus nerve. As shown in **Figure 1**, the response potentials were observed at the stimulation currents of 0.3, 0.5, and 1 mA. The latency of the action potentials was about 18 ms.

### Vagus Nerve Stimulation Ameliorates the Severity in Caerulein-Induced Acute Pancreatitis

**Figure 2:**
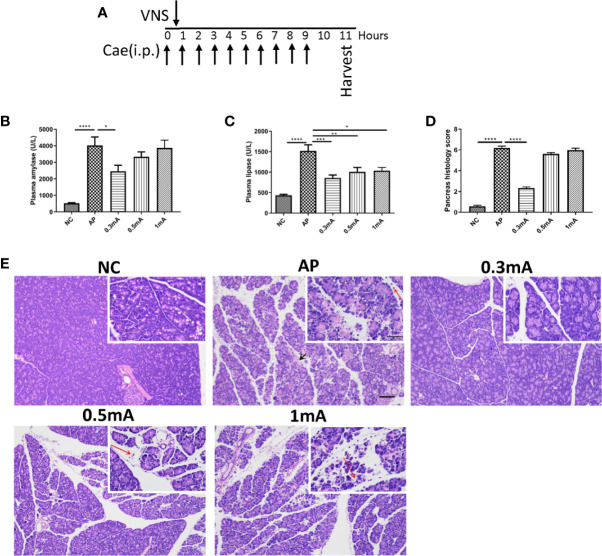
Vagus nerve stimulation alleviates the severity of caerulein-induced pancreatitis. Different intensities of VNS (0.3, 0.5 and 1mA currents) were applied to pancreatitis mice 30 min after first i.p. caerulein injection. Samples were collected 2 h after the last caerulein injection. **(A)** Schematic description of study design and experimental timeline. **(B)** Plasma amylase. **(C)** Plasma lipase. **(D)** Pancreas histological score. **(E)** Representative H&E staining of pancreas sections. Black arrow represents pancreas necrosis and red arrow represents infiltrating cells. Scale bars: 100 μm; 50 μm (inset). Values are shown as means ± SE. n = 6 per group. *p < 0.05, **p < 0.01, ***p < 0.001, ****p < 0.0001. VNS vagus nerve stimulation, Cae caerulein, i.p. intraperitoneal, NC normal control, AP acute pancreatitis.

Pilot experiments were performed to determine the optimal current intensity of VNS in caerulein-induced AP animals. Four groups of animals were given VNS at graded levels of 0 mA (sham VNS, AP group), 0.3, 0.5, and 1.0 mA after the first caerulein injection. Plasma amylase and lipase levels and morphological changes of pancreas which characterize the severity of AP were measured. The current of 0.3 mA dramatically suppressed plasma amylase (2455.0 ± 367.5 vs. 4024.0 ± 511.8 U/L, *p* < 0.05) and lipase (858.9 ± 71.3 vs. 1518.0 ± 149.3 U/L, *p* < 0.001) elevations compared with AP mice (**Figures 2B, C**). In addition, decreases in inflammatory cells infiltration and tissue edema were observed in pancreas of this group (**Figures 2D, E**). Therefore, for all later experiments, the stimulation intensity was set to 0.3 mA.

### Vagus Nerve Stimulation Suppresses TNF-α and Upregulates Pancreatic and Splenic Percentages of α7nAChR^+^ Macrophage in Caerulein-Induced Pancreatitis

**Figure 3:**
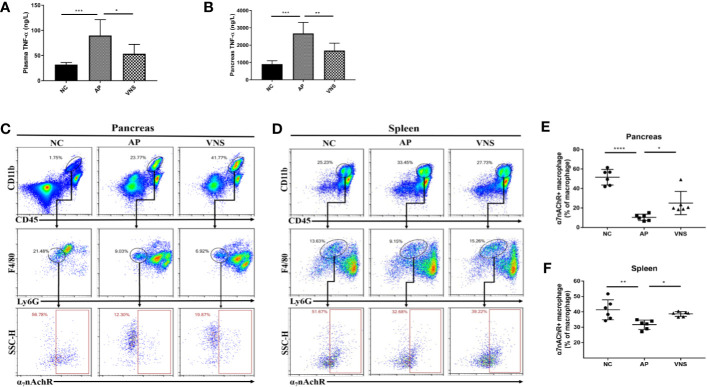
Vagus nerve stimulation decreases TNF-α levels and increases the frequency of α7nAChR^+^ macrophages in caerulein-induced pancreatitis. AP was induced by 8 hourly i.p. injection of caerulein and VNS was applied to mice 30 min after the first injection. Mice were harvested 2 h after the last injection. **(A)** Plasma TNF-α levels. **(B)** Pancreas TNF-α levels. Pancreatic and splenic leukocytes were isolated for flow cytometry. Leukocytes were gated (CD11b^+^CD45^+^). Macrophages were gated (CD11b^+^CD45^+^F4/80^+^Ly6G^−^) and analyzed for α7nAChR positive cell frequency. The percentages and representative flow cytometry plots illustrating α7nAChR^+^ macrophages in the pancreas **(C, E)** and the spleen **(D, F)**. Data are shown as the means ± SE of 6 mice per group. *p < 0.05, **p < 0.01, ***p <; 0.001, ****p < 0.0001. VNS vagus nerve stimulation, NC normal control, AP acute pancreatitis.

Inflammation is a key component of AP. In particular, TNF-α exerts a critical impact on recruitment of inflammatory cells in the pathogenesis of AP. Thus, plasma and pancreatic TNF-α levels were assessed. As shown in **Figures 3A, B**, caerulein-induced AP led to elevated circulating and pancreatic TNF-α concentrations, while stimulation of the left cervical vagal nerve abrogated these effects.

As CAP is crucial in mediating the effects of VNS and monocytes/macrophages are major sources for TNF-α, we further assessed the expression of α7nAChR on pancreatic and splenic macrophages (CD45^+^CD11b^+^F4/80^+^Ly6G^−^ cells). For NC mice, 51.5 ± 3.3% of macrophages in the pancreas and 41.3 ± 2.6% of macrophages in the spleen expressed α7nAChR. For AP mice, α7nAChR^+^ macrophage decreased to 10.4 ± 1.3% in the pancreas (*p* < 0.0001) and 31.6 ± 1.2% in the spleen (*p* < 0.01). VNS treatment significantly increased α7nAChR^+^ macrophage in the spleen and pancreas (**Figures 3C–F**).

Taken together, these findings indicate that VNS treatment results in anti-inflammatory effects in the caerulein-induced AP model and suggest a possible regulatory role of α7nAChR^+^ macrophage in inflammation suppression.

### Splenocytes From Vagus Nerve Stimulation Treated Mice Confer Protection Against Caerulein-Induced Pancreatitis

**Figure 4:**
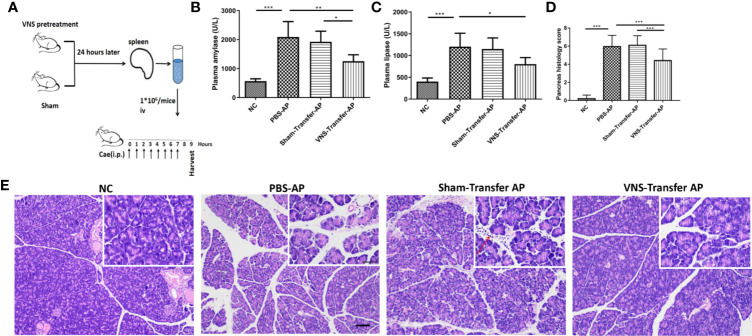
Adoptive transfer of splenocytes from VNS-treated mice protects against pancreatitis in naive recipient mice. **(A)** Donor mice underwent VNS or sham treatment. Twenty-four h later, splenocytes isolated from donor mice were injected iv into recipient mice. Twenty-four h after splenocyte transfer, the recipient mice were subjected to caerulein-induced pancreatitis. Mice were harvested 2 h after the eighth caerulein injection. **(B)** Plasma amylase. **(C)** Plasma lipase. **(D)** Pancreas histological score. **(E)** Representative H&E staining of pancreas sections. Red arrow represents infiltrating cells. Scale bars: 100 μm; 50 μm (inset). Values are shown as means ± SE. n = 6. *p < 0.05, **p < 0.01, ***p < 0.001. VNS vagus nerve stimulation, iv intravenous, Cae caerulein, i.p. intraperitoneal, NC normal control, AP acute pancreatitis.

Spleen is a key component of the vagus mediated anti-inflammatory pathway. Our results also confirmed the upregulation of α7nAChR^+^ macrophage in the spleen after VNS. Therefore, adoptive transfer experiments were performed to investigate the relevance of spleen in the protective effect of VNS. As shown in **Figure 4A**, donor mice were treated with sham stimulation or VNS. Twenty-four h later splenocytes were isolated and transferred to the recipient mice. After another 24-h, caerulein-induced AP was made. Compared with PBS treated AP mice, adoptive transfer of splenocytes from sham-treated mice showed no changes in plasma amylase and lipase levels. Although massive infiltrating leukocytes were observed in Sham-transfer AP pancreas, pancreas histological score showed no statistical difference between Sham-transfer AP mice and PBS-AP mice. In contrast, adoptive transfer of splenocytes from VNS-treated mice provided protection against AP. As shown in **Figures 4B–E**, significant statistical differences of the plasma amylase level (1255 ± 90.0 vs. 1923 ± 149.3 U/L, *p* < 0.05) and pancreas histological score (4.4 ± 0.2 vs. 6.1 ± 0.1, *p* < 0.0001) were found in VNS-transfer group compared with Sham-transfer group. These data demonstrated splenocytes from VNS-treated mice is sufficient to protect against AP in recipient mice.

### Splenectomy Does Not Significantly Affect the Protective Effects of Vagus Nerve Stimulation on Caerulein-Induced Pancreatitis

**Figure 5:**
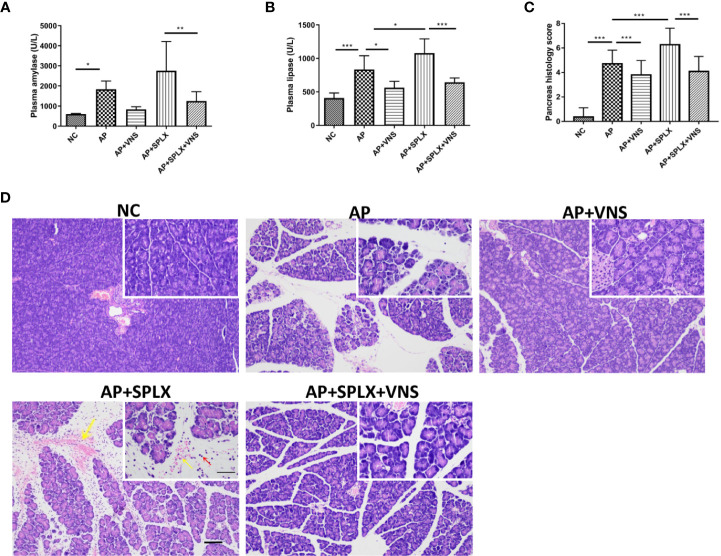
Vagus nerve stimulation protects against experimental pancreatitis independently of the spleen. Splenectomy or sham surgery was performed 5 days before VNS or sham treatment. AP was induced by 8 hourly i.p. injection of caerulein and VNS was applied to mice 30 min after the first injection. Mice were harvested 2 h after the last injection. **(A)** Plasma amylase. **(B)** Plasma lipase. **(C)** Pancreas histological score. **(D)** Representative H&E staining of pancreas sections. Yellow arrow represents pancreas hemorrhage and red arrow represents infiltrating cells. Scale bars: 100 μm; 50 μm (inset). Values are shown as means ± SE. n = 6. *p < 0.05, **p < 0.01, ***p < 0.001. VNS, vagus nerve stimulation; SPLX, splenectomy; NC, normal control; AP, acute pancreatitis.

To further confirm the role of spleen in the protective effect of VNS in AP, we performed experiments with mice subjected to splenectomy. Splenectomy significantly exacerbated the severity of AP, as evidenced by the increased plasma lipase level and pancreas histological damage (**Figures 5B–D**). As shown in **Figure 5D**, splenectomy resulted in massive pancreas hemorrhage and intensive inflammatory cell infiltrations. However, splenectomy failed to alter the beneficial effect of VNS on AP. No significant differences were detected in plasma amylase (832.9 ± 55.8 vs. 1252.0 ± 188.7 U/L, *p* > 0.05)/lipase (562.4 ± 38.9 vs. 644.1 ± 26.0 U/L, *p* > 0.05) levels or pancreas histological score (3.8 ± 0.1 vs. 4.0 ± 0.1, *p* >0.05) between AP+VNS group and AP+SPLX+VNS group. These findings show that splenectomy does not significantly affect the protective effect of VNS on the magnitude of pancreas inflammation.

### Vagus Nerve Stimulation Attenuates Tissue Damage and Prolongs Survival of Mice With PDL-Induced Pancreatitis

**Figure 6:**
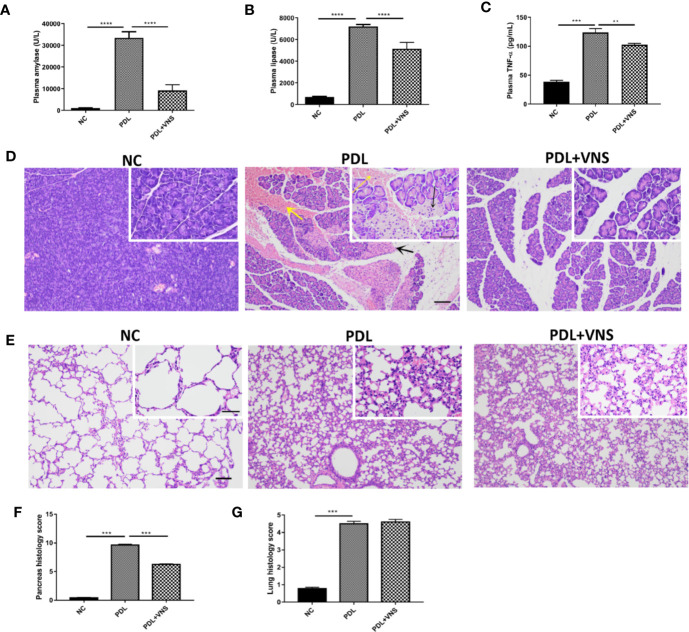
Vagus nerve stimulation alleviates pancreas injury and does not significantly affect pulmonary injury in pancreatic duct ligation-induced pancreatitis. VNS was applied to mice 30 min after PDL and samples were collected 12 h after PDL (n = 9). **(A)** Plasma amylase. **(B)** Plasma lipase. **(C)** Plasma TNF-α levels. **(D)** Representative H&E staining of pancreas sections. Black arrow represents pancreas necrosis and yellow arrow represents pancreas hemorrhage. Scale bars: 100 μm; 50 μm (inset). **(E)** Representative H&E staining of lung sections. Scale bars: 100 μm; 50 μm (inset). **(F)** Pancreas histological score. **(G)** Lung histological score. **p < 0.01, ***p < 0.001, ****p < 0.0001. VNS, vagus nerve stimulation; PDL, pancreatic duct ligation; NC, normal control.

To further verify the therapeutic effect of VNS, severe AP model caused by PDL was used. VNS dramatically suppressed plasma amylase (9266 ± 2522 vs. 33496 ± 2829 U/L, *p* < 0.0001), lipase (5159 ± 587.2 vs. 7207 ± 183.6 U/L, *p* < 0.0001) and TNF-α (102.6 ± 2.1 vs. 123.6 ± 6.8 pg/ml, *p* < 0.005) elevations as compared with PDL group (**Figures 6A–C**). Specifically, histological evaluation of H&E-stained pancreatic tissue suggested that PDL-induced AP showed diffuse edema, widespread occurrence of acinar necrosis, leukocyte infiltration and intensive hemorrhage. Only mild edema and leukocyte infiltration were found in VNS group (**Figure 6D**). Total pancreas histological score was significantly lower in VNS mice compared to PDL mice (**Figure 6F**). Acute lung injury is a common complication of severe AP. However, VNS exerted no protection against pancreatitis-associated lung injury, as demonstrated by pulmonary histological scores (**Figures 6E, G**).

**Figure 7:**
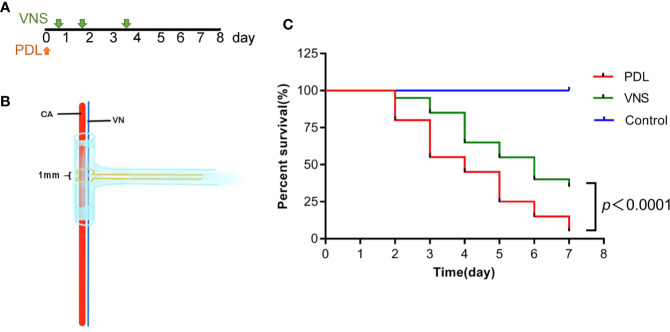
Vagus nerve stimulation prolongs survival of mice with pancreatic duct ligation-induced pancreatitis. **(A)** For survival studies, VNS was applied to PDL mice 30 min, 1 day and 3 days after surgery. **(B)** Cuff electrode was secured about the outside of the entire carotid sheath containing the vagus nerve. **(C)** Kaplan-Meier curve presents survival rate. n = 20 per group. VNS, vagus nerve stimulation; PDL, pancreatic duct ligation; CA, carotid artery; VN, vagus nerve.

VNS treatments were applied to PDL mice at 30min, 1 day and 3 days after modeling (**Figure 7A**). VNS was delivered through a bipolar cuff electrode (**Figure 7B**). As shown in **Figure 7C**, Kaplan-Meier curves revealed significant difference in survival rate between VNS and PDL mice (*p*<0.0001).

## Discussion

Due to the anti-inflammatory effects, VNS has become a major topic of interest in the treatment of several inflammatory diseases. In AP, previous animal studies suggested that vagotomy resulted in enhanced disease severity indicating innate physiologic role for vagus nerve in modulating disease (18). We here show, for the first time, that electrical stimulation of the left cervical carotid sheath containing the vagus nerve improved several disease-associated parameters in AP, including levels of plasma hydrolases and pancreas histologic abnormalities. In this study, the therapeutic effect of VNS was verified in two independent AP models: caerulein stimulation, which causes mild and reversible AP, and PDL, which causes severe hemorrhagic AP associated with substantial mortality.

Macrophages and neutrophils are pathogenic factors in AP. The inflammatory monocytes recruited into the pancreas were much greater than that of neutrophils during the acute phase of pancreatitis, which reflects the crucial role of infiltrated monocytes in the pathogenesis of early AP (17). Infiltrated monocytes at the local pancreas site are further activated into macrophages and the extent of activation determines the disease severity. Also, previous studies have described macrophage as an important parameter involved in the anti-inflammatory effect of VNS (11). It is commonly believed VNS activates macrophage *via* the α7nAChR, which leads to less production of pro-inflammatory cytokines. We found AP modeling resulted in a significant reduction of α7nAChR^+^ macrophages. In addition, α7nAChR^+^ macrophages were increased by VNS in both spleen and pancreas, indicating α7nAChR^+^ macrophages may play a crucial role in the protective effect of VNS. Splenocytes from VNS pretreatment mice also conferred protective effects in AP mice. These suggested that the CAP, which includes α7nAChR^+^ macrophages is activated by VNS. PDL-induced severe AP is associated with acute lung injury. In contrast to pancreas damage, the extent of pulmonary injury remained unchanged after VNS. This is in line with previous endotoxemia studies, which showed that VNS decreased TNF-α levels in the systemic compartment, the spleen, the liver, and the heart besides the lung (19). This is most likely associated with the unique features of alveolar macrophages, an anti-inflammatory phenotype but not susceptible to VNS, which are different from all other residential macrophages (19, 20).

Spleen plays a central role in the CAP for the neural control of the systemic inflammation (21). In models of acute kidney injury and colitis, the protective effects of VNS required the spleen (10, 22). Vagus nerve preganglionic nerons connect with the spleen through the sympathetic noradrenergic splenic nerve (23, 24). The activation of splenic nerve leads to norepinephrine releasing, which binds to β_2_-adrenergic receptors on splenic T cells. These T-cells are acting almost like neurons, releasing another neurotransmitter Ach and activating splenic macrophages through the α7nAChR, which results in reduction of pro-inflammatory cytokines release (12). However, we failed to attribute a crucial role to the spleen in the observed protective effect of VNS. For AP mice, whose spleens were removed, VNS could still dampen inflammation and decrease pancreatitis severity. This finding was consistent with the study focusing on vagus nerve and gastrointestinal inflammation. VNS reduced intestinal inflammation independent of the spleen while resident macrophages in small intestine were the ultimate target of the vagus nerve in the gut (25, 26). It is known that pancreas tissue is also vagally innervated. We therefore speculate that left cervical VNS could increase acetylcholine release from pancreas nerve endings and activate α7nAChR^+^macrophages in pancreas, thus causing an anti-inflammation effect. However, this requires further investigation.

VNS is emerging as a prospect way for treatment of inflammatory diseases. According, these is an interest to explore the methods for VNS in different experiment settings. Stimulation of the cervical vagus nerve under an ultrasound-guided needle electrode positioned at the carotid sheath in mice has been reported recently (27). As the visualized cervical vagus nerve in mice was very thin, we stimulated the entirety of the carotid sheath to avoid surgical injury of nerve. Using electrical stimulation of the left carotid sheath we activated the vagus nerve as evidenced by evoked compound action potentials from the subdiaphragmatic vagus nerve. One limitation of the study was that we stimulated the entire carotid sheath. Aside from the vagus nerve, the carotid sheath also contains the carotid sinus, which has also been shown to exert anti-inflammatory effects in LPS-induced systemic inflammation when stimulated (28). We performed an additional experiment to differentiate the effect of stimulating the nerve from stimulating other components of the carotid bundle. We found that electrical stimulation of the left carotid sheath as well as the isolated left cervical vagus nerve protected against AP. However, if the vagus nerve was cut, the protective effect by electrical stimulation of the left carotid sheath was abrogated (**Supplementary figure 1**). These data indicate that the effects seen in this study were due to the stimulation of the vagus nerve itself.

In summary, VNS reduced inflammation and pancreas injury in both caerulein and PDL induced AP; it also increased survival from PDL AP. The anti-inflammatory effect of VNS in AP might be related to increased α7nAChR expression on macrophage and spleen is not a central effector organ. Thus, VNS may be useful as a novel anti-inflammatory strategy to treat AP.

## Data Availability Statement

All datasets presented in this study are included in the article/**Supplementary Material**.

## Ethics Statement

The animal study was reviewed and approved by Institutional Animal Care and Use Committee of Nanjing University of Chinese Medicine.

## Author Contributions

All authors contributed to the study conception and design. Animal operations and flow cytometry were performed by ZWu and LZ. Pathology evaluation was performed by QY and ZWa. Data collection and analysis were performed by ZT and LZ. The first draft of the manuscript was written by LZ and all authors commented on previous versions of the manuscript. All authors contributed to the article and approved the submitted version.

## Funding

This work was supported by the National Natural Science Foundation of China (81704164).

## Conflict of Interest

The authors declare that the research was conducted in the absence of any commercial or financial relationships that could be construed as a potential conflict of interest.

## Supplementary Material

Supplementary Figure 1 Protection against caerulein-induced pancreatitis by electrical stimulation of the left carotid sheath is dependent on the vagus nerve. Acute pancreatitis (AP) was induced by 10 hourly i.p. of 100 μg/kg of caerulein. VNS was performed by stimulation of the left carotid sheath (VNScs) or direct stimulation of the left cervical vagus nerve (VNSd) half an hour after the first injection of caerulein. Vagotomy (VGX) was performed as follows: Half an hour after the first caerulein injection, the left carotid sheath was exposed and the left cervical vagus nerve was ligated and transected. After vagotomy, electrical stimulation or sham stimulation (0mA) was applied to the left carotid sheath. **(A)** Plasma amylase. **(B)** Plasma lipase. **(C)** Representative H&E staining of pancreas sections. Scale bars: 100 μm and 50 μm (inset) **(D)** Pancreas histological score. **p* < 0.05, ***p* < 0.01, *****p* < 0.0001.

## Abbreviations

Ach, Acetylcholine; AP, Acute pancreatitis; CAP, Cholinergic antinflammatory pathway; i.p., Intraperitoneal injection; NC, Normal control; PDL, Pancreatic duct ligation; SPLX, Splenectomy; VNS, Vagus nerve stimulation; α7nAChRs, α7 nicotinic acetylcholine receptors.
