# Supplementary figures and images for: Vagus Nerve Stimulation Decreases Pancreatitis Severity in Mice
Source: Front Immunol. 2021 Jan 14;11:595957. doi: 10.3389/fimmu.2020.595957 (PMC7840568; doi:10.3389/fimmu.2020.595957)

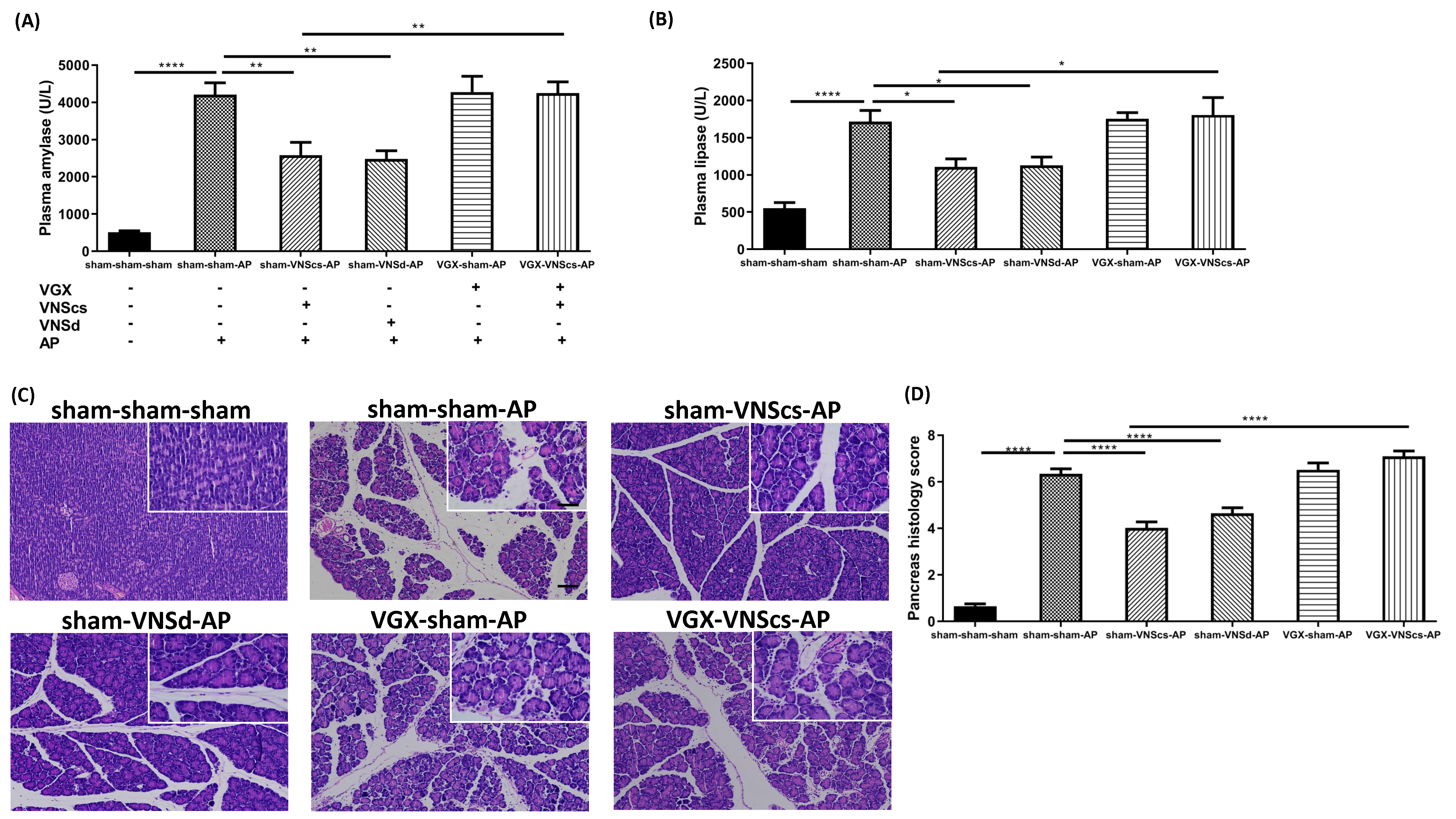

Supplement: Supplementary Figure 1 — Protection against caerulein-induced pancreatitis by electrical stimulation of the left carotid sheath is dependent on the vagus nerve. Acute pancreatitis (AP) was induced by 10 hourly i.p. of 100 μg/kg of caerulein. VNS was performed by stimulation of the left carotid sheath (VNScs) or direct stimulation of the left cervical vagus nerve (VNSd) half an hour after the first injection of caerulein. Vagotomy (VGX) was performed as follows: Half an hour after the first caerulein injection, the left carotid sheath was exposed and the left cervical vagus nerve was ligated and transected. After vagotomy, electrical stimulation or sham stimulation (0mA) was applied to the left carotid sheath. (A) Plasma amylase. (B) Plasma lipase. (C) Representative H&E staining of pancreas sections. Scale bars: 100 μm and 50 μm (inset) (D) Pancreas histological score. *p < 0.05, **p < 0.01, ****p < 0.0001. [file Image_1.tiff]
